# Evaluation of Agile Team Work Quality

**DOI:** 10.1007/978-3-030-58858-8_11

**Published:** 2020-08-18

**Authors:** Alexander Poth, Mario Kottke, Andreas Riel

**Affiliations:** 6 IT University of Copenhagen, Copenhagen, Denmark; 7 University of British Columbia, Vancouver, BC Canada; 8 Volkswagen AG, Berliner Ring 2, 38436 Wolfsburg, Germany; 9 Grenoble INP, G-SCOP, CNRS, Grenoble Alps University, 38031 Grenoble, France

**Keywords:** Agile team work quality (aTWQ), Large-scaling agile, Quality assurance (QA), Agile transformation

## Abstract

The maturity of organizations is measured with process assessment models like the ISO/IEC 33001. The product quality is aligned with internal and external product quality charactersitics based on models like the ISO/IEC 25010. With the shift from the Tailorism-driven process orientation to a more people centric organization, the two dimensions process and product quality have to be extened by the people or team quality dimension. The presented approach offers aspects for agile Team Work Quality (aTWQ), as well as related measurement indicators. The approach is evaluated in the large enterprise context of the Volkswagen AG. The indicators of aTWQ have been integrated and established in the agile tool box for a sustainable agile transition of the company.

## Introduction

Several big enterprises like Cisco [1], Ericsson [2], and Volkswagen [3] are in the process of agile transformation. Accompanying tools and measures have to scale from individual project teams to bigger organizational entities [4]. The key of agile development is the team who delivers the customer value. However, systematic approaches to team development in software developing industries are rare. They need to cover criteria for the determination of team culture and performance, metrics, as well as recommendations for improvement. In this article, we present the aTWQ (agile Team Work Quality) approach to supporting teams in improving their agile mindset and practices by themselves without external assessments. Given the legislative and cultural context that is typical for large European enterprises, aTWQ shall meet the following particular requirements and constraints:

- The approach shall not use specific roles that are typically fulfilled by a particular person to avoid individual performance measures to be aligned with workers council mindset in enterprises.
- The approach shall be appropriate for integration in project and program reviews to measure transition progress from a governance perspective.
- The approach shall be applicable as a self-service by the teams to ensure scaling without centralized coaching etc. and support the autonomy of the teams during evolving.

The lean and agile approaches most frequently used in industry, Scrum and SAFe®, do not address TWQ explicitly. In SAFe®, one of the four core-values is “Build-in Quality” [5]. In the deep dive documentation [6], however, the focus is product quality and “Flow” as a generic construct for all other aspects of quality. The process quality is implicitly addressed by links to other topics. TWQ is not mentioned at all, and therefore implicit. On the other hand, the consequence of this observation is: everything that is needed for quality is done inherently and not defined in SAFe®. In Scrum, the heart of the value creation is the team, which is supported by the Definition of Done (DoD) for achieving product quality, as well as the team retrospectives for process improvement. The team itself does not get any kind of explicit quality-related instructions and tasks. Instead, the daily, open communication and commitments are essential parts of TWQ. This is motivated by the aspects like mutual trust and performance monitoring which are observed in [7]. Also in [8] aspects like the ability to complete whole tasks or feedback are shown to have an impact to the team work quality. In [9] it is observed that team work quality correlates with performance in some settings which is an important fact for organization development. Also, collocation and diversity in teams [10] helps to improve team work quality.

The particular challenge related to TWQ is the fact that TWQ is part of internal quality aspects that are typically hidden and invisible from the outside. This makes it difficult in lean and agile environments to identify and explicitly “spend effort” on them. The ISO/IEC 25010:2011 makes this more transparent by distinguishing “quality in use” from “product quality”. The latter is often directly addressed by regulation and compliance requirements like security or reliability. The process quality is treated as a “first class citizen”, because there are powerful and influential (external) stakeholders for legal compliance. Therefore, without some explicit measures and metrics related to TWQ, a systematic development is difficult from the organizational point of view.

## A Team-Based Approach to Agile TWQ

Team work aspects have been treated to a large extent in literature, e.g. [11] and [12]. Some of this previous work addresses agile team work quality explicitly [13] or [14] some also propose organizational models fostering team work quality [15]. During the design of our approach, we focused on integration of different concepts with a longer evaluation time to not have the work to start from scratch and get benefits form the diversity of the different approaches we are integrating. The three approaches we consider most relevant are the Team Work Quality (TWQ) [14], Team Climate Inventory (TCI) [16] and Group Development Questionnaire (GDQ) [15] because they address both the team development and maturity. The TWQ approach focuses on quality indicators of team work. The TCI approach developed over years and evaluates team indicators related to the teams’ working structures for innovation. The GDQ approach focuses on evaluating the teams’ alignment with stages of group development. Based on [17], the following empirical observations provide the basis of our aTWQ approach:

- Team Performance is based on TWQ.
- TWQ and the TCI have similar “content”.
- TCI works well with GDQ.

Based on [14] and [18], we derived the initial team-level approach covering the six aspects communication, coordination, balance of contribution, mutual support, effort, cohesion. These six quality aspects lead to team performance [19], legitimating economically the effort for measurement and further TWQ improvement. We combined these aspects with those of TCI and defined 19 related questions to come up with a holistic team evaluation questionnaire for aTWQ, see Table 1.

**Table 1.** aTWQ questionnaire with specific indicators for Scrum and SAFe® and team development level.

Topic	Question (Base Practices)	Scrum	SAFe®	Level
Participative safety	Do we have a “**we** are in it together” **attitude** *driven by the* ***ability and willingnes****s to help and support each other in carrying out their tasks?*			IV
	Do people keep **each other informed** about work-related issues in the team *supported by a frequent communication?*	Daily Scrum	Program, team backlog	I
	Do people **feel understood** and accepted by one another?			III
	Are there real attempts to **share information** throughout the team driven *by openness of the information exchange?*	Daily Scrum, Retrospective	Portfolio Kanban, Inspect & Adapt	(I) III
	Is there a lot of **give and take** *by the team members’ motivation to maintain the team?*		Innovation and Planning Iteration	IV
	Do we **keep in touch** with one another as a team *by accepting that team goals are more important than individual goals?*		Pairing/frequent review	III
Support for innovation	Is this team always moving towards the development of new answers?			IV
	Is this team open and responsive to change?	Inspect & Adaptation	Innovation and Planning Iteration	III
	Do people in this team always search for fresh, new ways of looking at problems?	Retrospective	Innovation and Planning Iteration, PI Planning	III
	Do members of the team provide and share resources to help in the application of new ideas driven *by team members’ ability and willingness to share workload?*	Inspect & Adaptation	Innovation and Planning Iteration	III
	Do team members provide practical support for new ideas and their application by *prioritize the teams’ task over other obligations*?	self-organizing	Innovation and Planning Iteration	IV
Vision	How clear are you about what your team’s objectives are?	(Product) Vision, Sprint Goal	Vision	I
	To what extent do you agree with these objectives?	Sprint commitment	PI planning	I
	To what extent do you think other team members agree with these objectives?	Refinement	ART commitment	I
	To what extent do you think members of your team are committed to these objectives?	Sprint commitment, DoD	ART commitment	I
Task orientation	Do your team colleagues provide useful ideas and practical help to enable you to do the job to the best of your abilities?		Pairing/frequent review	IV
	Are team members prepared to question the basis of what the team is doing?	Daily Scrum, Refinement		IV
	Does the team critically appraise potential weaknesses in what it is doing in order to achieve the best possible outcome?	Refinement, Retrospective		II
	Do members of the team build on one another’s ideas in order to achieve the highest possible standards of performance?	Refinement, Retrospective		(I) IV
**Coordination**	Is there a **common understanding** when working on parallel subtasks, and agreement on common work breakdown structures, schedules, budgets and deliverables?	Backlog, Stories	Roadmap, Portfolio, ART, Iteration plan, Stories	III

TWQ aspects not explicitly covered by the TCI questionnaire have been added and printed in italics. Terms printed in bold letters signify the most important aspects of the respective question. Column 3 and 4 show the mapping of the questions to Scrum and SAFe®, respectively, based on the specific approach’s elements covering the aspects addressed by the questions. Hence, the TCI/TWQ questions represent generic practices, while the associated elements from Scrum or SAFe represent specific practices of either approach. Both combined constitute the practice set of aTWQ in a specific team environment. The sparsely populated columns 3 and 4 indicate that neither Scrum nor SAFe® cover aTWQ aspects well. The indicators of the approaches are based on the current versions of SAFe® 5.0 and the Scrum Guide version of Nov. 2017.

For the integration into the project reviews [20] evaluating individual product teams, a group of teams (like programs), as well as entire organizational units, an extension beyond a typical team size is needed. For the context of aTWQ, a team is constituted by people who have common goals within a purpose. The team size is aligned with the agile definition of 7–9 individuals [21]. A group is a collection of people or teams coordinating outcomes and efforts.

In the aTWQ approach, the extension to groups larger than one team is realized with the Group Development Questionnaire (GDQ) because in scaling agile approaches there is no “one big team”. In SAFe®, for example, there exist different types of teams like the technical and business teams sharing a common basic approach. “Both types of teams strive for fast learning by performing work in small batches, assessing the results, and adjusting accordingly” [22]. This leads us to deriving that in SAFe, a group of different types of teams is managed. To handle this appropriately, something beyond TWQ is needed to show that the group which forms a SAFe® environment works fine.

The evaluation of the readiness of organizations is based on the spiral dynamics approach, which is usable in larger social systems like the GDQ. These two models provide the basis for using the aTWQ approach from individual teams to larger organizational units including many teams that work for some shared objectives. Based on this, the Level specification has been made in column 5 of Table 1. These levels represent the following GDQ approach stages: (I) Dependency and inclusion, (II) Counter-dependency and fight, (III) Trust and structure, and (IV) Work and productivity. The numbers in parentheses indicate the rating aligned without the mindset objective primarily based on the formal application of the respective agile aspects only. For example, in the *Scrum theater,* people apply some Scrum methods “mechanically” without actually forming a Scrum team with an agile mindset – this Scrum theater have to be rated with the parentheses level. The levels can be used by the teams to prioritize the improvement actions – start with actions on lower levels to establish a base to build on for higher level actions. The four maturity levels can be easily mapped to ratings used in specific process assessment frameworks such as the ISO/IEC 33001:2015. To have some specific indicators for the rating, column 3 and 4 can be used. Furthermore, the level rating is an indicator for the maturity of teams based on the TCI/GDQ approach.

## Evaluation and Improvement Iterations

In the first step, the initially designed approach was simulated with the coaches of the Agile Center of Excellence (ACE) [23] which are the Volkswagen Group IT competence center for agile transitions and quality experts from the Quality Innovation Network (QiNET) [24] which is an innovation network for IT quality within the Volkswagen AG. The simulation was realized by virtual application of the aTWO questionnaire to teams coached in the past. For each simulation a point in the past was used as timestamp for answering the aTWO questions based on the situation around the timestamp. During the simulation the answers of the teams were simulated by the coaches/experts based on their knowledge about the team. Based on the answers potential chances and risks for the team development were derived. Then the timestamp was move ahead to check if the chances or risks identified by the aTWO approach are realistic to validate the questionnaire as a starting point for team improvements. An initial Proof of Concept (PoC) was done in the Scrumban aligned product team of TaaS [25]. The self-assessments taken ca. 1.5 h. The team can answer the questions in a way it is most useful and common in the team – bullet points or phrases are valid options to document evidences and indicators as well as for improvement ideas. But it is important to make the rating in the defined NPLF-schema to be able to compare team ratings of different organizations.

Some facts about the TaaS PoC: The concerned service was introduced in 2016 and has been offered in the Volkswagen Group since 2017. Over the years, evolving the team constellations have led to an established devops team with end-to-end responsibly for the service delivery. In April 2020, the team included an internal product owner, two internal software engineers and one external software engineer with a primary focus on product development and third-level ops-support, as well as one external part-time devops engineer with primary focus on first and second-level support and some third-level support activities. The team members’ experience levels covers a wide range from junior developer to senior engineer. After a team composition change a few weeks earlier, the team was in a re-balancing phase. The application of the aTWQ questionnaire worked fine and was conducted as a dedicated task of a team retrospective. The identified enhancement potentials were used like retrospective outcomes and lead to actions for team improvement. Some small improvements based on the feedbacks and observations were made about aTWQ and are reflected in the version of Table 1. As an outcome, a spreadsheet was derived with supporting notes and remarks for the teams. This sheet is the core of the aTWQ self-service kit.

Team sizes and self-assessments were similar in the two other applications we investigated. The teams remained stable at least one year before the self-assessment was conducted. All these teams belong to the same organizational unit, which has approximately 25 employees. Furthermore, the organizational unit “shares” experts in the teams. Therefore, in each self-assessment of a team at least one person has two self-assessments. The organizational unit achieves a 2-digit million Euro turnover based on a service-catalog based delivery approach. The service delivery is realized with a few hundreds of external partners. The service are a full stack from management activates, consulting, coding to operations. The evaluation results from this application shows that the self-service kit is ready to use. This leads to the next step to reflect the aTWQ self-service kit in the coach guild of the Volkswagen AG and offer it to the coaches with all brands. In a final step, the integration into the agile tool box was made for a general availability to everybody in the Volkswagen AG. Furthermore, aTWQ was integrated into the *agile project review* [20] in June. This provides the base to compare teams and organizations in the future. To avoid that this approach is used only as a management tool the self-service kit offered to ensure that independent form external triggers the team can work in a safe private environment to improve them.

## Conclusion

With aTWQ, we proposed a model for the awareness of the team-dimension of the three quality dimensions product-, process- and team-quality. We specified an explicit indicator set for the most popular agile approaches Scrum and SAFe®. First evidences for relevance and added-value for effective team development in Scrumban environments have been given by the self-assessments and the derived team actions.

The key contributions *to theory* can be summarized by the identification of the gap between the current quality-models to the real world in industrial settings which emphasize agile team work which is not explicitly addressed and covered by the established product and process quality models and approaches. The identification of possible approaches reduced this gap by the integration of the TCI, TWQ and DGQ approach to the aTWQ approach with a focus on the application in real world product teams. The initial analysis about the state-of-the-art provides a basis for more sophisticated research about the added value created by the aTWQ approach in the context of team-, multi-team- and organizational-level.

The context of the development and evaluation of aTWQ is a large enterprise setting with a European culture and mindset. This narrows the possibilities and degrees of freedom by design. The evaluation criteria in the questionnaire are not fine grained which lets room for interpretation of what is adequate if no explicit evidences are expected and no indicators are given by the evaluation model. Currently aTWQ has an open design to leave the decision by the teams in case of self-application and by the reviewer from the governance in case of “external” team evaluations. The interpretation by a more or less constant governance reviewer team will give sufficient comparability between the teams within an organization. Really mature agile teams will actively request for external “feedbacks” to get the ranking to other teams and learn from external inspiration for their improvement journey. This kind of limitation is a chance by design to ensure continuous improvement within the teams and organizations because they have not static target like an evidence or indicator list which have to be fulfilled and the *“aTWQ story is done”.*
